# Exploratory Analysis of the Digital Health Gender Paradox in Consumer-Facing Digital Health Technology Adoption: Cross-Sectional Survey Study

**DOI:** 10.2196/101963

**Published:** 2026-09-29

**Authors:** Jessica Kathleen Wallace, Mehak Batra, Tafheem Ahmad Wani, Michael Liem, Kylie Ovenden, James Boyd, Therese Keane, Urooj Raza Khan

**Affiliations:** 1 School of Psychology and Public Health La Trobe University Bundoora, Victoria Australia; 2 School of Education La Trobe University Bundoora, Victoria Australia

**Keywords:** digital health, gender differences, health equity, digital health literacy, technology adoption, Capability, Opportunity, Motivation – Behavior, COM-B model, mobile health, mHealth, telehealth, artificial intelligence, AI, chatbots

## Abstract

**Background:**

Digital health technologies have become integral to modern health care, yet consumer adoption remains uneven. These technologies span a range of consumer-facing tools, including remote care services, mobile apps, wearable devices, conversational agents, and digital medication services, with uptake varying considerably across tool types and by gender. An earlier analysis of this dataset revealed a gender paradox: women reported higher digital literacy compared to men yet demonstrated lower adoption and curiosity toward digital health tools. The Capability, Opportunity, Motivation – Behavior (COM-B) model proposes that behavior occurs when individuals have the capability (skills and confidence), opportunity (access, affordability, and contextual support), and motivation (trust, perceived usefulness, and willingness) to act.

**Objective:**

This study aimed to examine associations between digital literacy, self-reported COM-B domains, gender, and adoption across digital health technologies and to explore whether these associations differ by gender.

**Methods:**

This cross-sectional quantitative study with an integrated qualitative component enrolled 416 adults and used multivariable logistic regression to examine associations between COM-B domains and adoption across five consumer-facing digital health technologies. Exploratory gender-by-predictor interaction and gender-stratified analyses examined potential gender-specific patterns, while self-reported reasons for nonuse were mapped to COM-B domains using directed content analysis.

**Results:**

Women had lower odds of chatbot/virtual assistant use (adjusted odds ratio [aOR] 0.32, 95% CI 0.18-0.59), marginally lower odds of e-pharmacy use (aOR 0.48, 95% CI 0.22-1.01), and higher odds of mobile health (mHealth) app use (aOR 2.59, 95% CI 1.28-5.25). Of 20 exploratory interaction tests, chatbot digital literacy-by-gender (*P*=.021) and capability-by-gender (*P*=.044) interactions were nominally significant. In directed barrier analysis, the gender distribution differed significantly for mHealth apps (*P*=.009), whereas the wearable result showed a trend (*P*=.079); motivation-related barriers were most frequent for most technologies.

**Conclusions:**

Behavioral determinants of digital health technology adoption varied by technology type. Women used several technologies less often than men despite reporting higher digital literacy, capability, and motivation, and opportunity emerged as the most consistent factor across the models examined. Exploratory analyses suggested these patterns may differ by gender for selected technologies but did not support a single gender-differentiated pathway. These findings are exploratory and require confirmation in larger studies.

## Introduction

### Background

Health systems worldwide are under escalating pressure from aging populations, rising chronic disease prevalence, and persistent workforce shortages [1,2]. In response, many countries are accelerating digital transformation to support more sustainable, person-centered models of care. As digital health technologies become embedded in routine health care delivery, attention has increasingly shifted to digital health readiness, including the data, technology, analytic, workforce, and human factors required to realize the benefits of digital health, while minimizing harms [2].

Internationally, a broad range of digital health technologies, such as telehealth, mobile health (mHealth) apps, wearable devices, conversational agents, and e-pharmacies, are being integrated into health care systems. However, adoption remains uneven and is shaped by stakeholder needs, implementation context, trust, infrastructure, and alignment with health care workflows [3,4]. Evidence from Australia reflects a similar pattern: although telehealth use surged during the COVID-19 pandemic, adoption of newer or more autonomous tools has been slower, particularly among older adults, people with lower educational attainment or income, those living in rural or remote areas, and individuals with limited digital or health literacy [5,6]. Accordingly, Australian digital health policy identifies inclusive, person-centered engagement as central to realizing the benefits of digital transformation across the population [7].

### Prior Digital Health Technology Adoption/Implementation Research

Research examining digital health technology adoption has expanded rapidly in recent years; however, important limitations remain. Much of the existing literature focuses on single technologies, applies narrow or technology-specific behavioral models, or targets specific subpopulations, such as older adults or individuals with chronic disease [8-10]. In addition, although behavioral determinants are increasingly recognized as important to digital health technology adoption and implementation, they are not always examined using structured behavioral frameworks, and limited attention has been given to how these determinants vary across gender groups or across different types of digital health technologies [8,10,11].

Australian research shows that digital health technology adoption is strongly patterned by socioeconomic status, education, broadband availability, and digital health literacy, which together predict engagement, particularly with national personal health records [12,13]. Consistent with this, implementation reviews continue to identify persistent adoption gaps in rural and underserved populations and note the limited use of behavioral theory in digital health technology adoption research [14]. More broadly, this literature often emphasizes individual digital literacy, while giving less attention to the structural and contextual conditions that shape engagement with digital technologies [4]. The World Health Organization (WHO) Global Strategy on Digital Health similarly notes that literacy alone is insufficient for equitable participation without inclusive design, supportive infrastructure, and equitable access [15].

Together, these gaps mean the evidence base provides an incomplete account of why adoption varies across population groups. In particular, gender is often reported descriptively rather than examined as a factor that may shape how individuals experience, interpret, and engage with digital health technologies. This study analysed behavioral drivers across multiple digital health technologies and examined gender differences in adoption using a structured behavioral framework.

### Gendered Patterns of Digital Engagement

A preliminary conference paper using this dataset reported high-level descriptive findings on digital literacy, the current use of digital health technologies, and curiosity toward emerging technologies. The analysis observed that women report higher digital literacy but lower use of some digital health technologies; however, it did not formally examine the behavioral mechanisms underlying these differences using multivariable or Capability, Opportunity, Motivation – Behavior (COM-B)–based analyses [16]. Digital literacy refers to the skills and confidence required to access, understand, and use digital technologies effectively [13,17]. Curiosity, defined as an intrinsic motivation to seek information and explore technologies, is conceptually distinct from digital literacy or confidence [18,19]. This study applied the COM-B framework to examine behavioral determinants of adoption through multivariable regression, gender-by-predictor interaction analyses, gender-stratified models, exploratory nested modeling, and COM-B-guided analysis of reported reasons for nonuse. In this way, the current study moved beyond describing gender differences in literacy and technology use to investigate whether capability, opportunity, and motivation are associated with the observed gender-specific patterns of adoption.

Global analyses highlight persistent gender inequalities in digital participation. Even where women attain high levels of education and digital skills, engagement with digital technologies remains constrained by affordability, safety concerns, time poverty, and structural inequalities in science, technology, engineering, and mathematics (STEM) education [20-23]. Time poverty, limited discretionary time arising from disproportionate domestic and caregiving responsibilities, further restricts opportunities for digital engagement [24].

Consistent with these broader patterns, Australian and international studies have documented a gender gap in digital health engagement. Women, particularly in their roles as patients and carers, may be more likely to experience inequitable or suboptimal digital health interactions [25,26]. Scholars also caution that digital health tools may insufficiently reflect women’s needs and that design choices, data governance practices, or algorithms can embed gender bias, while limited integration of gender-responsive and intersectional perspectives in digital health research may reinforce existing inequalities [25,27].

These dynamics are particularly significant, given women’s central roles within health systems. In Australia, women make up a large proportion of the formal health workforce, and primary informal carers are also predominantly female [28,29]. Qualitative evidence from Australia also shows that many women use digital health information, not only as patients, but also to provide advice and support to family members and friends [30]. Consequently, women’s engagement with digital health technologies is consequential in its own right and may shape broader patterns of adoption, trust, and diffusion within families and communities [23,30].

Overall, this evidence points to a potential capability-opportunity mismatch in digital health technology adoption: women’s digital skills and familiarity with health care settings may coexist with structural, temporal, and contextual barriers that constrain opportunities to use digital technologies [24,25,31]. Addressing these dynamics is essential to ensure that digital transformation does not widen existing inequalities and to support the design and development of technologies that are responsive to the needs, preferences, and contexts of diverse populations [25,32].

### Theoretical Framework: COM-B and the Behavior Change Wheel

To examine these dynamics, this study applied the COM-B model, which conceptualizes behavior as the product of interaction between capability, opportunity, and motivation [33]. Capability refers to the psychological and physical capacity to perform a behavior (including relevant knowledge and skills). Opportunity captures external factors, both physical and social, that enable or prompt the behavior. Motivation encompasses reflective and automatic processes, including goals, beliefs, emotions, and habits, that energize and direct behavior [33].

COM-B forms the hub of the Behavior Change Wheel (BCW), a widely used framework for diagnosing behavioral barriers and mapping them to potential intervention strategies and policy levers [33]. Relative to technology adoption models, such as the Technology Acceptance Model and the Unified Theory of Acceptance and Use of Technology, which emphasize constructs such as perceived usefulness, ease of use, performance expectancy, and facilitating conditions [34,35], COM-B provides a broader behavioral lens by incorporating psychological, social, and environmental determinants of behavior. In this study, however, the COM-B constructs were operationalized through self-reported composite indices and should be interpreted as perceived behavioral domains rather than objective measures of physical or psychological capability.

### Study Aim

This study aimed to examine gender differences in digital health technology adoption and to explore how self-reported behavioral determinants within the COM-B model are associated with those patterns. The analyses were exploratory and association based; they were not designed to establish causal pathways or mediation.

Using a survey dataset, this study examined technology-specific associations with capability, opportunity, and motivation and evaluated exploratory gender-by-predictor interactions. It addressed gaps in multitechnology behavioral research, while explicitly distinguishing overall pooled predictors from evidence of gender moderation. To translate behavioral insights into practical implications, findings were mapped to the BCW, which linked COM-B determinants to intervention functions and policy strategies that may support more equitable digital health technology adoption. As this was a self-reported study, results reflect perceived rather than objectively measured behaviors; nonetheless, they provide theory-informed insights into gendered determinants of digital health engagement that can inform equitable digital health policy, design, and implementation.

## Methods

### Overview

A cross-sectional online survey (Research Electronic Data Capture [REDCap]) was conducted to examine Australian adults’ digital literacy, engagement with digital health technologies, and preferences for future services. Eligible participants were aged ≥18 years and residing in Australia. As an exploratory pilot examining behavioral pathways and gender differences, no a priori sample size calculation was performed. The achieved sample (N=416) supported multivariable and interaction analyses but may have been underpowered to detect small effects.

The survey was developed collaboratively by academic investigators, Medibank Private Limited, and consumer organizations to ensure relevance and accessibility. It included demographic items and open- and closed-ended questions on digital health experiences and behaviors.

Participants were recruited using convenience sampling through social media, consumer advocacy networks, Medibank communication channels, and research team networks. The sample was not designed to be nationally representative. Data were collected between July 21 and August 24, 2023. Participants could enter a prize draw for an AU $50 (US $36) voucher funded by Medibank Private Limited.

Survey design was informed by the COM-B model and the Australian Digital Capability Framework, with items adapted to the digital health context (Multimedia Appendix 1) [36]. A review by the Consumer Health Forum of Australia and state-based organizations was conducted to optimize accessibility.

### Measurement

#### Predictors

Digital literacy was measured using a 22-item scale covering information/data literacy, communication/collaboration, content creation, safety, and problem solving. Items were averaged, with higher scores indicating greater self-reported digital competence (α=0.81).

Capability, opportunity, and motivation were operationalized as theory-informed composite indices based on COM-B-informed survey sections. Capability reflected perceived functional readiness, knowledge, skills, and confidence; opportunity reflected perceived access, affordability, availability, infrastructure, and social/professional support; and motivation reflected trust, perceived usefulness and benefits, preferences, interest, intentions, and emotional responses to digital technology. Digital literacy was treated as a related but distinct measure of self-reported digital competence. All measures were self-reported and therefore reflected perceived rather than objectively assessed capability or digital skills.

The Capability index comprised 15 items (α=0.83), the Opportunity index 5 items (α=0.76), and the Motivation index 13 items (α=0.61). These indices were developed for this exploratory study and were not intended as validated psychometric COM-B scales. Items were coded so that higher scores represented greater capability, opportunity, or motivation, with negatively worded items reverse-coded, as appropriate. Item scores were averaged within each domain and standardized (*z*-scores), such that odds ratios represented a 1-SD increase. Exploratory factor analyses indicated that the Opportunity index showed the clearest evidence of unidimensionality, consistent with its internal consistency estimate. The Capability and Motivation indices showed evidence of multidimensionality, suggesting they each capture related but heterogeneous constructs within their respective domains; this is reflected in the lower internal consistency of the Motivation index in particular and should be considered when interpreting findings associated with these composites. Intercorrelations between digital literacy and the three COM-B indices were modest to moderate (r=0.39-0.57), providing preliminary evidence that the constructs were related but distinct.

Complete item-to-domain and subdomain mapping, scoring rules, reverse-coded items, handling of “do not know” responses, item-level missingness, and exploratory measurement analyses are provided in Tables S2-S4 in Multimedia Appendix 2.

#### Outcomes (Behavior)

Adoption of each technology (telehealth, mHealth apps, wearable devices, chatbots/virtual assistants, and e-pharmacy) was operationalized as self-reported current use and coded as a binary outcome (current user vs nonuser). This measure captured current user status rather than the frequency, duration, or intensity of technology use.

#### Effect Modifier

Gender (female vs male) was examined as an effect modifier. Interaction terms between gender and each of the four predictors (digital literacy, capability, opportunity, and motivation) were included in separate exploratory models (Table S1 in Multimedia Appendix 2), resulting in 20 interaction tests across five technology outcomes. These analyses were hypothesis generating rather than confirmatory.

#### Covariates

Participants were grouped by age (≤34, 35-54, and ≥55 years) to reduce sparse cells and maintain adequate group sizes. The exact age in years was not collected; therefore, age could not be modeled as a continuous variable. Models adjusted for age group, education (low/intermediate, bachelor/advanced diploma, postgraduate), occupation (white collar, blue collar/service, other/not in labor force), region (Eastern, Central/Western, Southern/Territory), and residence (metropolitan vs rural/remote). Occupation and region were collapsed from the original survey categories to maintain model parsimony. These groupings were defined during data preparation, and covariates were selected a priori as structural determinants of digital access.

### Statistical Analysis

#### Overview

Quantitative responses, including Likert-scale items, were summarized using descriptive statistics. Qualitative free-text responses were analyzed using directed content analysis guided by the COM-B framework. Cross-tabulations by key demographic variables, including age, gender, education level, and geographic location, were performed using IBM SPSS Statistics, whereas all inferential and multivariable analyses were conducted in Stata version 19.5 [37].

#### Quantitative Analysis

Participant characteristics were summarized by gender. Categorical variables were compared using *χ*² tests. Continuous or ordinal variables were summarized as median (IQR) and compared using Mann-Whitney *U* tests. Differences in adoption rates by gender were compared using *χ*² tests.

Logistic regression models were used to examine associations between digital health technology adoption and gender, digital literacy, COM-B domains, and sociodemographic covariates. The primary fully adjusted models included gender, digital literacy, capability, opportunity, motivation, age, education, occupation, region, and rural/remote residence. Adjusted odds ratios (aORs) with 95% CIs were reported. Digital literacy, capability, opportunity, and motivation were standardized (*z*-scores), such that their aORs represented a 1-SD increase.

To descriptively examine changes in the gender coefficient after inclusion of the COM-B domains, pre-COM-B-reduced models included gender, digital literacy, and the same sociodemographic covariates as the primary models, but excluded capability, opportunity, and motivation. Covariate definitions and coding were identical between these models and the primary fully adjusted models. These comparisons were descriptive and were not interpreted as evidence of mediation or mechanisms.

Effect moderation by gender was explored by including gender-by-predictor interaction terms for digital literacy, capability, opportunity, and motivation across each of the five technology outcomes, resulting in 20 interaction tests. These analyses were exploratory and hypothesis generating. For nominally significant interactions, adjusted predicted probabilities of technology adoption were estimated at −1 SD, the mean, and +1 SD of the relevant predictor, holding other covariates at their observed values using Stata’s *margins* command. Gender-stratified logistic regression models were additionally estimated to characterize within-gender patterns (Table S1 in Multimedia Appendix 2).

Multicollinearity was assessed using variance inflation factors (<2). Model fit was evaluated using likelihood-ratio *χ*² and pseudo-R² statistics. Robust (Huber-White) SEs were used for the primary models. There were no missing data for variables included in the primary multivariable models; consequently, all models included the full analytic sample (N=416).

Given the relatively small number of nonevents for some outcomes, model complexity and robustness were additionally assessed. For each primary model, we examined the number of complete cases, events/nonevents, and estimated parameters. Simplified sensitivity models retained gender, digital literacy, capability, opportunity, motivation, and age, while excluding the remaining sociodemographic covariates. These were fitted for both the primary models and the 20 gender-by-predictor interaction models and compared with the primary fully adjusted models to assess sensitivity to model complexity. A further sensitivity analysis excluding participants in the “other/not in labor force” occupation category was directionally consistent with the primary models (results not shown).

Given the lower internal consistency of the Motivation index (α=0.61), additional sensitivity analyses examined alternative motivation specifications, including exclusion of the e-pharmacy-specific preference item and a reduced composite excluding the three items with the lowest corrected item-rest correlations (Table S5 in Multimedia Appendix 2). Given the exploratory nature of this pilot study and multiple behavioral predictors across technology-specific models, estimates were interpreted cautiously, with emphasis on the magnitude, direction, consistency, and uncertainty of associations.

#### Qualitative Analysis

For participants who had not adopted specific technologies, reasons for nonuse were captured through open-ended responses and analyzed using directed content analysis guided by the COM-B framework. Responses were imported into Stata version 19.5 and preprocessed prior to coding. Initial categorization was conducted using lexical pattern matching (*regexm* functions) to flag responses corresponding to capability, opportunity, and motivation domains (eg, “no access,” “prefer in-person,” “not confident”). Lexical matching was used as an initial screening and organizational step rather than as the basis for final classification. The technology-specific coding framework, operational definitions, and illustrative lexical triggers are provided in Table S6 in Multimedia Appendix 2.

Two PhD-qualified researchers independently reviewed all flagged responses against the complete response text and validated or revised the initial classifications based on contextual meaning. Where responses reflected more than one COM-B construct, a primary barrier was assigned according to the predominant reason for nonuse identified from the complete response. Discrepancies between researchers were resolved through iterative discussion and consensus. Because the two researchers’ independent judgments were not retained as separate ratings, an interrater agreement statistic was not calculated. Coding rigor was instead supported by the technology-specific framework with documented operational definitions (Table S6 in Multimedia Appendix 2). Coding was blinded to participant gender; gender was linked to the final COM-B classifications only after coding for the gender-stratified descriptive analyses.

Within-gender frequencies of capability, opportunity, and motivation barriers were summarized for each technology and integrated descriptively with the quantitative findings. Because open-ended responses were obtained from a predefined survey sample rather than through iterative qualitative sampling, saturation was not used as a stopping criterion. Given small cell counts in the gender-stratified barrier analyses, Fisher’s exact test was used for comparisons.

### Ethical Considerations

Ethics approval was obtained from the La Trobe University Low-Risk Human Research Ethics Committee (HREC #23100). Participation was voluntary, with informed consent obtained prior to survey completion. Data were collected anonymously using REDCap, a secure, web-based platform designed to support data collection and management for research. De-identified data were exported and stored securely on a password-protected research drive, with access restricted to the research team.

## Results

### Participant Profile

Of 416 respondents, 61.1% (n=254) were male and 38.9% (n=162) female. The age distribution differed by gender (*P*=.019), with women more often aged ≥35 years (n=84, 51.8%) and more likely to hold postgraduate qualifications (n=73, 45.1%) compared to men (*P*=.001). Occupational distribution differed significantly between genders, with approximately three-quarters (n=123, 75.9%) of women working in white-collar roles compared to 42.9% (n=109) of men (*P*<0.001), whereas men were more commonly employed in blue-collar or service occupations. Residence area was broadly similar between genders, although regional distribution differed significantly.

Across COM-B domains, women reported higher digital literacy, capability, and motivation, whereas opportunity scores were similar between genders (Table 1). These differences supported examining gender-specific pathways in subsequent analyses.

**Table 1:** Participant characteristics by gender (N=416).

Characteristics			Male (n=254)		Female (n=162)		*P* value	
**Age group (years), n (%)**								.019^a^
	≤34	158 (62.2)		78 (48.2)		—^b^		
	35-54	68 (26.8)		59 (36.4)		—		
	≥55	28 (11.0)		25 (15.4)		—		
**Education level, n (%)**								<.001^a^
	Low/intermediate	26 (10.2)		19 (11.7)		—		
	Bachelor/advanced diploma	155 (61.0)		70 (43.2)		—		
	Postgraduate	73 (28.7)		73 (45.1)		—		
**Occupation category, n (%)**								<.001^a^
	White collar	109 (42.9)		123 (75.9)		—		
	Blue collar/service	143 (56.3)		23 (14.2)		—		
	Other^c^	2 (0.8)		16 (9.9)		—		
**Residence area, n (%)**								.261^a^
	Metropolitan	180 (70.9)		123 (75.9)		—		
	Rural/remote	74 (29.1)		39 (24.1)		—		
**Region group, n (%)**								<.001^a^
	Eastern	202 (79.5)		100 (61.7)		—		
	Central/western	44 (17.3)		46 (28.4)		—		
	Southern/territory	8 (3.2)		16 (9.9)		—		
Digital literacy, median (IQR); minimum, maximum			3.2 (1.1); 2.1, 4.0		3.6 (0.5); 1.9, 4.0		<.001^d^	
Capability index, median (IQR); minimum, maximum			3.0 (0.7); 2.1, 3.8		3.3 (0.5); 2, 3.7		<.001^d^	
Opportunity index, median (IQR); minimum, maximum			3.4 (0.6); 1.6, 4.0		3.4 (1.0); 1, 4.0		.372^d^	
Motivation index, median (IQR); minimum, maximum			3.4 (0.3); 2.8, 3.8		3.5 (0.2); 2.6, 3.8		<.001^d^	

^a^*χ*² test.

^b^Not applicable.

^c^Not in the labor force.

^d^Mann-Whitney *U* test (statistically significant results at *P*<.05).

### The Adoption Paradox: Technology-Specific Gender Gaps

Overall, nearly all participants (n=413, 99.3%) reported using at least one digital health technology. Adoption was highest for clinician-mediated modalities, including telehealth and mobile apps.

Gender differences were observed for newer or more autonomous technologies. Women were less likely to use wearable devices, chatbots/virtual assistants, and e-pharmacies (Table 2). After full adjustment, women remained significantly less likely to use chatbots/virtual assistants, whereas the association with e-pharmacy use was marginal and the association with wearable device use was attenuated (Table 3). These findings indicate that gender differences are technology-specific, particularly for tools requiring greater autonomy or trust.

**Table 2:** Uptake of digital health technologies by gender.

Technology and use			Total participants (N=416), n (%)		Male (n=254), n (%)		Female (n=162), n (%)
**Telehealth (*P*=.059^a^)^b^**							
	Yes	385 (92.5)		240 (94.5)		145 (89.5)	
	No	31 (7.5)		14 (5.5)		17 (10.5)	
**mHealth^c^ app (*P*=.44)^b^**							
	Yes	323 (77.6)		194 (76.4)		129 (79.6)	
	No	93 (22.4)		60 (23.6)		33 (20.4)	
**Wearable device (*P*<.001^d^)^b^**							
	Yes	329 (79.1)		225 (88.6)		104 (64.2)	
	No	87 (20.9)		29 (11.4)		58 (35.8)	
**Chatbot/virtual assistant (*P*<.001^d^)^b^**							
	Yes	308 (74.0)		222 (87.4)		86 (53.1)	
	No	108 (26.0)		32 (12.6)		76 (46.9)	
**e-Pharmacy (*P*<.001^d^)^b^**							
	Yes	357 (85.8)		235 (92.5)		122 (75.3)	
	No	59 (14.2)		19 (7.5)		40 (24.7)	

^a^*P*=.05-.10 (trend).

^b^*χ*² test.

^c^mHealth: mobile health.

^d^Statistically significant results at *P*<0.05.

**Table 3:** Multivariable logistic regression models^a^ of predictors of digital health technology adoption by gender and COM-B^b^ domain.

Predictor^c^	Telehealth, aOR^d^ (95% CI), *P* value	mHealth^e^ app, aOR (95% CI), *P* value	Wearable device, aOR (95% CI), *P* value	Chatbot/virtual assistant, aOR (95% CI), *P* value	e-Pharmacy, aOR (95% CI), *P* value
Gender (1=female)	1.51 (0.58-3.95), .403	2.59 (1.28-5.25), .008^f^	0.61 (0.33-1.12), .113	0.32 (0.18-0.59), <.001^f^	0.48 (0.22-1.01), .052^g^
Digital literacy (*z*-score)	0.47 (0.24-0.92), .026^f^	0.74 (0.42-1.29), .288	0.70 (0.40-1.21), .199	0.88 (0.54-1.44), .613	0.84 (0.50-1.38), .487
Capability index (*z*-score)	0.88 (0.44-1.73), .703	1.44 (0.81-2.58), .215	0.77 (0.47-1.26), .315	0.56 (0.35-0.89), .016^f^	0.70 (0.43-1.15), .158
Opportunity index (*z*-score)	1.80 (1.15-2.83), .010^f^	3.18 (2.11-4.77), <.001^f^	1.93 (1.33-2.79), <.001^f^	1.16 (0.84-1.59), .380	1.26 (0.89-1.76), .184
Motivation index (*z*-score)	1.50 (0.92-2.45), .101	0.93 (0.70-1.25), .627	1.02 (0.72-1.45), .914	1.26 (0.93-1.70), .135	1.50 (1.05-2.15), .026^f^

^a^Each model was adjusted for age, education, occupation, region, and rural/remote residence.

^b^COM-B: Capability, Opportunity, Motivation – Behavior.

^c^Predictors were standardized (*z*-scores), except gender, so each odds ratio represents the change in odds for a 1-SD increase in the predictor. Gender was entered as a binary categorical variable and was not standardized.

^d^aOR: adjusted odds ratio.

^e^mHealth: mobile health

^f^Statistically significant results at *P*<.05.

^g^*P*=.05-.10 (trend).

### Behavioral Determinants of Digital Health Technology Adoption (COM-B Domains and Gender)

Before inclusion of the COM-B domains, pre-COM-B-reduced models, including gender, digital literacy, and sociodemographic covariates, showed significantly lower odds of wearable-device (aOR 0.47, 95% CI 0.26-0.85; *P*=.012), chatbot/virtual assistant (aOR 0.30, 95% CI 0.16-0.54; *P*<.001), and e-pharmacy (aOR 0.41, 95% CI 0.20-0.86; *P*=.018) use among women. Gender was not associated with telehealth (aOR 1.01, 95% CI 0.42-2.44; *P*=.981) or mHealth app (aOR 1.36, 95% CI 0.72-2.56; *P*=.342) use. After inclusion of capability, opportunity, and motivation in the primary fully adjusted models (Table 3), the magnitude of these gender associations changed to varying degrees.

Opportunity emerged as the most consistent and influential determinant of digital health technology adoption across technologies. A 1-SD increase in opportunity was associated with higher odds of telehealth, mHealth app, and wearable device use, highlighting the central role of access, affordability, availability, and contextual support in enabling engagement.

Motivation showed a nominally significant positive association with e-pharmacy adoption in the primary model; however, this association should be interpreted cautiously, as it was attenuated and the CI included the null when model complexity was reduced in sensitivity analyses. This pattern may potentially reflect the role of trust and perceived safety in medication-related digital transactions but requires confirmation in larger studies. In contrast, digital literacy showed an inverse association with telehealth use and was not independently associated with other technologies after adjustment, suggesting that technical competence alone is insufficient to drive adoption.

Capability was negatively associated with chatbot/virtual assistant use, indicating that individuals with higher perceived capability were less likely to engage with conversational agents. This may reflect a preference among more capable users for alternative sources of information or care.

After full adjustment, women remained significantly less likely to use chatbots and marginally less likely to use e-pharmacies, while being 2.6 times more likely to use mHealth apps. Collectively, these results indicate that structural and contextual opportunity is the most consistent determinant of engagement across digital health technologies, with patterns of adoption varying by technology type and gender (Table 3).

The primary pooled models examined overall associations between behavioral determinants and technology adoption across the full sample, adjusted for gender and sociodemographic covariates. These overall associations should be distinguished from the exploratory gender moderation analyses presented in the *Gender Moderation and Stratified Behavioral Pathways* section. All primary models included 416 complete cases and 14 predictor parameters. The numbers of events/nonevents were 385/31 for telehealth, 323/93 for mHealth app use, 329/87 for wearable device use, 308/108 for chatbot use, and 357/59 for e-pharmacy use. Simplified-model sensitivity analyses showed that the principal COM-B findings were generally directionally consistent, particularly the opportunity associations with telehealth, mHealth app, and wearable-device use and the capability association with chatbot use. For e-pharmacy use, the motivation association remained positive but was attenuated in the simplified model (aOR 1.35, 95% CI 0.98-1.87; *P*=.070) compared to the primary model (aOR 1.50, 95% CI 1.05-2.15; *P*=.026).

### Gender Moderation and Stratified Behavioral Pathways

Exploratory interaction analyses suggested potential gender differences in behavioral associations, most notably for chatbot/virtual assistant use. Nominal interactions were observed for digital literacy (interaction aOR 2.10, 95% CI 1.12-3.96; *P*=.021) and capability (interaction aOR 1.83, 95% CI 1.02-3.29; *P*=.044), as shown in Table S1 in Multimedia Appendix 2. Adjusted predicted probabilities further illustrated these patterns. For capability, the adjusted predicted probability of chatbot use decreased from 92.1% at −1 SD to 70.7% at +1 SD among men and from 67.5% to 55.5% among women, suggesting a steeper decline among men. For digital literacy, the adjusted predicted probability of chatbot use decreased from 89.0% to 76.4% among men but increased from 55.8% to 64.8% among women across the same range.

Interaction estimates were directionally consistent across specifications. In simplified models retaining only the primary predictors and age, chatbot interactions were similar but more precise (digital literacy aOR 2.29, 95% CI 1.28-4.07; capability aOR 1.99, 95% CI 1.17-3.39), and the wearable capability interaction reached nominal significance (aOR 1.82, 95% CI 1.05-3.18) having been nonsignificant under full adjustment (aOR 1.63, 95% CI 0.89-2.99). These models omit education, occupation, region, and rural/remote residence and are reported only as a check on model complexity. The interaction findings are hypothesis generating.

Gender-stratified analyses provided additional descriptive evidence of potentially differing behavioral patterns. Opportunity was positively associated with mHealth app adoption in both genders. For wearable devices, opportunity was positively associated with adoption among both women and men, while higher capability was associated with lower adoption among men. For chatbot use, higher capability was associated with lower adoption among men, whereas no statistically significant association was observed among women. These stratified findings were interpreted descriptively and not as independent evidence of gender differences.

### Gendered Barriers to Digital Health Technology Adoption (COM-B Mapping)

Reported barriers were consistent with the COM-B framework (Table 4). A detailed summary of self-reported barriers to digital health technology adoption by COM-B domain is provided in Multimedia Appendix 3, whereas Table 4 provides a brief overview. Motivation-related barriers, such as preference for in-person care or low perceived need, were the most commonly reported category overall, accounting for 41.5% (34/82; mHealth apps) to 69.6% (32/46; e-pharmacy) of nonuse reasons across technologies, and were the leading barriers for telehealth, chatbots/virtual assistants, and e-pharmacy. For wearable devices, opportunity-related barriers were the most frequent (n=36, 46.2%), and for mHealth apps, motivation- and capability-related barriers were equally common (n=34, 41.5% each). Capability-related barriers were otherwise less frequent, ranging from 6.5% (3/46; e-pharmacy) to 21.0% (21/100; chatbots/virtual assistants), whereas opportunity-related constraints, such as limited access, cost, and lack of availability, varied by gender and technology.

**Table 4:** Self-reported reasons for nonuse of digital health technologies by gender, mapped to COM-B^a^ domains.

Technology	Nonusers with a codable reason, n (%)	Opportunity, n/N (%)^b^	Motivation, n/N (%)^b^	Capability, n/N (%)^b^	*P* value^c^
Telehealth	30 (96.8)	M^d^: 4/14 (28.6); F^d^: 6/16 (37.5)	M: 8/14 (57.1); F: 10/16 (62.5)	M: 2/14 (14.3); F: 0	.460
mHealth^e^ app	82 (88.2)	M: 5/55 (9.1); F: 9/27 (33.3)	M: 22/55 (40.0); F: 12/27 (44.4)	M: 28/55 (50.9); F: 6/27 (22.2)	.009
Wearable device	78 (89.7)	M: 8/25 (32.0); F: 28/53 (52.8)	M: 12/25 (48.0); F: 22/53 (41.5)	M: 5/25 (20.0); F: 3/53 (5.7)	.079^f^
Chatbot/virtual assistant	100 (92.6)	M: 7/28 (25.0); F: 16/72 (22.2)	M: 14/28 (50.0); F: 42/72 (58.3)	M: 7/28 (25.0); F: 14/72 (19.4)	.755
e-Pharmacy	46 (78.0)	M: 5/16 (31.3); F: 6/30 (20.0)	M: 11/16 (68.8); F: 21/30 (70.0)	M: 0; F: 3/30 (10.0)	.464

^a^COM-B: Capability, Opportunity, Motivation – Behavior.

^b^Percentages are within-gender column percentages.

^c^Fisher’s exact test.

^d^M: male; F: female.

^e^mHealth: mobile health.

^f^*P*=.05-.10 (trend). *P*<.05 indicates a statistically significant gender difference. Totals differ from nonuser counts in Table 2 because barrier analyses included only nonusers who provided a codable reason for nonuse.

Women more frequently reported opportunity-related barriers for mHealth apps, including issues of access, affordability (“too expensive”), and contextual support (eg, “never been offered”), whereas men more commonly cited capability-related barriers, including uncertainty and technical skill limitations (“not sure how to use it”). Similar, but nonsignificant, patterns were observed for wearable devices, with women emphasizing access and affordability (n=28, 52.8%), including cost (“too expensive”) and lack of device ownership (“don’t have a smartwatch”), and men citing technical skill limitations (20%). For telehealth, chatbots, and e-pharmacies, motivational barriers such as low perceived usefulness (“no need”) and preference for in person care (“prefer human”) predominated for both genders, although opportunity related constraints were also evident, particularly for chatbots (“haven’t come across any”) and e-pharmacies (“not offered,” “haven’t had the chance”).

These findings suggest that structural barriers, such as cost, limited access, and lack of availability, were more prominent among women for selected technologies, most clearly mHealth apps, where the gender difference in the barrier profile was statistically significant. For mHealth apps, men more frequently reported capability-related barriers, including uncertainty and technical skill limitations. These qualitative findings complemented, but did not fully mirror, the quantitative results. In the quantitative models, higher capability was sometimes associated with a lower likelihood of use among men, particularly for chatbots and wearable devices. This divergence may reflect selective nonadoption among more confident users who perceive limited value in specific technologies. Meanwhile, the pooled quantitative models identified opportunity as an important overall correlate of adoption across several technologies. Taken together, these findings suggest technology-specific opportunity constraints rather than a consistently female-specific opportunity effect across all technologies.

## Discussion

### Principal Findings

This study applied the COM-B framework to examine gender differences in digital health technology adoption [33]. Although women reported higher digital literacy, capability, and motivation than men, they demonstrated lower unadjusted adoption of several technologies, particularly wearables, chatbots, and e-pharmacies. After adjustment, the most robust gender difference remained for chatbot/virtual assistant use, with a marginal difference observed for e-pharmacy use. These findings contribute to understanding the digital health gender paradox and suggest that lower engagement among women is unlikely to reflect a simple deficit in digital literacy, capability, or willingness to use these technologies. Rather, they are consistent with evidence that gendered digital health inequities may arise through system design, access, governance, and service contexts, rather than individual skill deficits alone [25,38].

The principal contribution of these findings is not simply that opportunity was an important overall predictor of adoption but also that the relationships between behavioral determinants and adoption appeared to vary according to both gender and technology type. Women’s lower unadjusted use of several technologies occurred despite their higher reported digital literacy, capability, and motivation, indicating that greater perceived capacity or readiness to use digital technologies does not necessarily translate into greater engagement. Exploratory interaction and gender-stratified analyses further suggested gender-differentiated associations for selected technologies, particularly chatbot/virtual assistant use. These findings extend the digital health gender paradox by suggesting that the behavioral conditions associated with adoption may differ across gender groups, although these patterns were not uniform across technologies and should be regarded as hypothesis generating rather than evidence of a single gender-specific mechanism.

An important distinction arising from these findings is between the capacity to use a digital health technology and actual engagement with that technology. Digital literacy and perceived capability reflect whether an individual has, or perceives themselves to have, the skills and confidence required to engage, whereas adoption also depends on whether appropriate opportunities for use are available and whether the technology is perceived as relevant or worthwhile. In this study, higher reported digital literacy among women did not consistently translate into greater technology use. This finding aligns with emerging evidence that, although necessary, digital literacy alone is insufficient to drive adoption in the absence of supportive structural conditions [12,13,15,39]. Effective digital health technology use requires not only skills and confidence but also access to technologies, integration into care pathways, and trust in how technologies are designed and delivered. Interventions focused solely on improving digital literacy are therefore likely to have limited impact unless accompanied by strategies that address opportunity-related barriers. It is important to note though that digital literacy and perceived capability are conceptually distinct from actual engagement. Having the capacity or confidence to use a technology does not necessarily mean that it is accessible, affordable, relevant, trusted, integrated into care, or ultimately used. Infodemiological research using Baidu search data similarly illustrates how observable population-level health information-seeking behavior can reveal patterns of digital engagement that are not equivalent to self-reported knowledge, capability, or intentions [40,41]. Although these studies do not directly examine the gender paradox investigated here, they provide a complementary example of the distinction between perceived capacity and observable digital behavior.

Opportunity emerged as one of the most consistent determinants of adoption across several technologies, particularly telehealth, mHealth apps, and wearable devices, reinforcing the importance of structural and contextual factors in enabling digital health engagement. However, the gender-specific pattern was more nuanced. In the self-reported barrier data, women more frequently identified opportunity-related constraints for some technologies, most clearly for mHealth apps, including barriers relating to access, affordability, and whether digital options were available or offered. A similar pattern was observed for wearable devices, although the evidence was less certain. Men, in contrast, more frequently reported capability-related barriers for some technologies. Together with the interaction and stratified analyses, these findings suggest potentially different behavioral patterns across gender groups, while also demonstrating that these patterns depend on the specific technology being considered. The opportunity-telehealth association should be interpreted with particular caution, given the small number of telehealth nonusers (n=31) relative to model parameters, which may contribute to imprecision in this estimate.

Alternative explanations should also be considered. The technologies examined differ substantially in purpose, cost, availability, required device ownership, integration with health care services, and the degree of autonomy required from users. Gender differences in health needs, prior exposure to particular technologies, device ownership, age distribution, occupation, or patterns of health care use may therefore also contribute to the observed adoption differences. In particular, the substantial difference in occupational composition between genders, with approximately three-quarters of women in white-collar roles compared with 43% of men, may have contributed to observed adoption patterns, given that white-collar occupations may involve greater prior exposure to digital technologies; although occupation was included as a covariate in all models, residual confounding from unmeasured aspects of occupational digital exposure cannot be excluded. Although the multivariable analyses adjusted for several sociodemographic characteristics, residual confounding and unmeasured contextual factors cannot be excluded.

### Unexpected Findings

After adjusting for COM-B domains, women were more likely than men to adopt mHealth apps. This pattern may indicate that women’s engagement with mHealth apps becomes more evident once structural and contextual factors are considered. Given that opportunity was the strongest determinant of mHealth app adoption, the result is consistent with mobile app engagement being associated not only with individual literacy or motivation but also with contextual conditions, such as access, availability, affordability, and service integration. However, because the study was cross-sectional, changes in gender coefficients across adjusted models should not be interpreted as evidence that these factors causally explain the gender difference. This interpretation aligns with prior research showing that digital health engagement is influenced by structural, service, access, and support-related conditions, even when digital literacy is present [13,25,39].

An inverse association between capability and chatbot adoption was observed among men. This may reflect selective nonuse among digitally confident individuals who perceive limited value, reliability, or usefulness in automated health agents. Emerging evidence highlights the role of trust, perceived usefulness, and user concerns in the adoption of artificial intelligence (AI)–enabled health chatbots, including concerns about accuracy, cybersecurity, empathy, and preference for human interaction [42-44].

Motivation showed a positive association with e-pharmacy adoption in the primary model; however, this association was attenuated and the CI included the null in the simplified sensitivity model. This finding should therefore be interpreted with caution and regarded as hypothesis generating. It may potentially reflect the role of trust and perceived safety when technologies involve medication management or financial transactions, consistent with prior research showing that perceived risk, trust, privacy, price, availability, and product-related concerns influence consumers’ willingness to purchase medicines online [45,46]; however, confirmation in larger, adequately powered studies is needed.

### Addressing Opportunity Barriers in Digital Health Technology Adoption

The distinction between capacity and engagement has practical implications for intervention design. Where opportunity-related barriers were observed in this study, particularly barriers concerning access, affordability, availability, and the offering of digital options, strategies directed solely at increasing digital skills are unlikely to address the full range of conditions associated with nonuse. Instead, implementation approaches may also need to modify the environments in which digital health technologies are made available and offered to consumers. The BCW proposes that opportunity-related barriers can be addressed through intervention functions, such as environmental restructuring and enablement, which modify the context in which behaviors occur [33,47]. Interventions most directly aligned with barriers reported in this study could therefore include improving access to appropriate technologies, reducing financial or device-related barriers, and ensuring that suitable digital options are consistently offered within health care encounters. In the context of digital health, this may include integrating digital services into clinical workflows; proactively offering digital options during health care encounters; and reducing financial, technological, or service access barriers that limit engagement, as suggested by broader implementation literature [7,15,48]. Communication strategies may further support adoption by increasing awareness, improving acceptability, and normalizing digital health services. Patient portal implementation provides a directly relevant example. In a multi-institutional patient portal implementation, uptake was shaped not only by patient interest or capability but also by provider engagement, change management support, leadership endorsement, broad rollout and marketing strategies, technology infrastructure, and policy alignment [49]. Similarly, a systematic review of patient portal adoption found that adoption rates were substantially higher in actively recruited controlled studies than in real-world deployments, suggesting that availability alone is insufficient unless implementation creates practical opportunities for use [50]. These examples illustrate how service design and implementation can influence the practical opportunity to engage with digital services, even among individuals who may already possess the skills required to use them. These findings should also be interpreted within broader gender inequalities in digital participation. Although women increasingly achieve high levels of education and digital skills, structural disparities persist in areas such as access to technology, participation in the digital workforce, and representation in technology development. These factors may influence how digital technologies are designed, implemented, and experienced by users [21,24,51]. Evidence from Australia similarly highlights the importance of gender diversity and inclusion in the digital health workforce, particularly for ensuring that digital health innovation reflects diverse perspectives and needs [38].

Taken together, these findings suggest that digital health technology adoption should not be conceptualized solely as an issue of individual digital literacy. Rather, adoption is shaped by structural opportunity, including access to services, integration into health care pathways, and the broader social context in which digital technologies are developed and deployed. Without interventions that expand opportunity and embed digital services within routine care, digital health initiatives risk reinforcing existing gender inequities despite high levels of individual capability and motivation.

### Strengths and Implications

The integration of quantitative modeling with self-reported barrier data strengthened interpretation by allowing behavioral associations identified quantitatively to be considered alongside participants’ reported reasons for nonuse. The inclusion of five different digital health technologies was also valuable because it demonstrated that adoption determinants were not uniform across technology types. Digital literacy levels were relatively high within the sample, providing a useful context in which to examine why comparatively high self-reported literacy and capability did not consistently correspond with adoption. Future research should examine longitudinal adoption patterns and evaluate interventions targeting opportunity-related barriers.

### Limitations

The cross-sectional study design limits causal inference and does not capture longitudinal changes in digital health technology adoption. Measures of digital literacy and technology use were self-reported and may therefore be subject to recall or social desirability bias. The study used convenience sampling through social media, consumer advocacy networks, Medibank communication channels, and research team networks; therefore, the sample may be subject to selection bias and should not be considered nationally representative of Australian adults. The binary adoption measure did not capture the frequency, duration, or intensity of technology use and may therefore represent different levels of engagement across the five technologies.

The sample was moderately skewed toward men (61.1% male), which may reflect the recruitment channels used and may limit generalizability. The high uptake observed, with 99.3% of respondents reporting use of at least one digital health technology, suggests that the sample may have included individuals with relatively high baseline engagement in digital health. This may limit the extent to which findings apply to populations with lower digital access, lower digital literacy, or less prior exposure to digital health technologies. Although this imbalance may have reduced statistical power to detect smaller gender-by-COM-B interaction effects, analyses were adjusted for key sociodemographic variables and supplemented with exploratory gender-stratified models.

### Conclusion

This study provided behavioral evidence on the digital health technology adoption gender paradox, showing that women’s lower unadjusted engagement with several digital health technologies occurs despite higher reported digital literacy, capability, and motivation. Using the COM-B framework, the findings suggest that behavioral determinants of adoption may vary by gender and technology type, with opportunity-related barriers, including access, affordability, and inconsistent offering of digital services, appearing particularly relevant to women’s nonuse for some technologies. Some findings also suggested that men’s nonuse may be more closely linked to capability-related concerns, including confidence and perceived ease of use. These gendered behavioral patterns suggest that digital transformation initiatives focused primarily on improving digital literacy may not achieve equitable adoption. Addressing structural opportunity barriers through gender-responsive, theory-informed implementation strategies may therefore be important for supporting equitable digital health technology adoption.

## Appendix

Multimedia Appendix 1 Survey instrument.

Multimedia Appendix 2 Gender interaction and stratified models.

Multimedia Appendix 3 Self-reported barriers to digital health technology adoption.

## Abbreviations

AI: artificial intelligence
aOR: adjusted odds ratio
BCW: Behavior Change Wheel
COM-B: Capability, Opportunity, Motivation – Behavior
mHealth: mobile health
REDCap: Research Electronic Data Capture

## Data Availability

The datasets generated and analyzed during the study are not publicly available due to ethics and privacy restrictions associated with participant consent and de-identified survey data but are available from the corresponding author upon reasonable request and subject to ethics approval requirements. For enquiries relating to the research presented in this manuscript, please contact URK.

## References

[1] (2025). Ageing and health. World Health Organization.

[2] Organisation for Economic Co-operation and Development (2023). Health at a Glance 2023: OECD Indicators.

[3] Shaw T, McGregor D, Brunner M, Keep M, Janssen A, Barnet S (2017). What is eHealth (6)? Development of a conceptual model for eHealth: qualitative study with key informants. J Med Internet Res.

[4] Van Velthoven MH, Cordon C (2019). Sustainable adoption of digital health innovations: perspectives from a stakeholder workshop. J Med Internet Res.

[5] Savira F, Orellana L, Hensher M, Gao L, Sanigorski A, Mc Namara K, Versace VL, Szakiel J, Swann J, Manias E, Peeters A (2023). Use of general practitioner telehealth services during the COVID-19 pandemic in regional Victoria, Australia: retrospective analysis. J Med Internet Res.

[6] (2023). Auditor-general report no. 10 2022-23: expansion of telehealth services (performance audit). Australian National Audit Office.

[7] (2023). National digital health strategy 2023-28. Australian Digital Health Agency (ADHA).

[8] Hepburn J, Williams L, McCann L (2025). Barriers to and facilitators of digital health technology adoption among older adults with chronic diseases: updated systematic review. JMIR Aging.

[9] Krahe MA, Larkins SL, Adams N (2024). Digital health implementation in Australia: a scientometric review of the research. Digit Health.

[10] Patel SB, Iqbal FM, Lam K, Acharya A, Ashrafian H, Darzi A (2025). Characterizing behaviors that influence the implementation of digital-based interventions in health care: systematic review. J Med Internet Res.

[11] Voorheis P, Bhuiya AR, Kuluski K, Pham Q, Petch J (2023). Making sense of theories, models, and frameworks in digital health behavior change design: qualitative descriptive study. J Med Internet Res.

[12] Alam K, Mahumud RA, Alam F, Keramat SA, Erdiaw-Kwasie MO, Sarker AR (2019). Determinants of access to eHealth services in regional Australia. Int J Med Inform.

[13] Cheng C, Gearon E, Hawkins M, McPhee C, Hanna L, Batterham R, Osborne RH (2022). Digital health literacy as a predictor of awareness, engagement, and use of a national web-based personal health record: population-based survey study. J Med Internet Res.

[14] Krahe MA, Baker S, Woods L, Larkins SL (2025). Factors that influence digital health implementation in rural, regional, and remote Australia: an overview of reviews and recommended strategies. Aust J Rural Health.

[15] (2021). Global strategy on digital health 2020-2025. World Health Organisation.

[16] Thanthiriwattage R, Liem M, Nouman M, Wani T, Marriner T, Ovenden K, Boyd J, Raza Khan U (2025). Australian healthcare consumers ‘curiosity’ in digital health technologies. Stud Health Technol Inform.

[17] McLean P, Oldfield J, Stephens A (2020). Draft Digital Literacy Skills Framework.

[18] Cervera RL, Wang MZ, Hayden BY (2020). Systems neuroscience of curiosity. Curr Opin Behav Sci.

[19] Kashdan TB, Gallagher MW, Silvia PJ, Winterstein BP, Breen WE, Terhar D, Steger MF (2009). The curiosity and exploration inventory-II: development, factor structure, and psychometrics. J Res Pers.

[20] (2017). Cracking the code: girls’ and women’s education in science, technology, engineering and mathematics. UNESCO.

[21] (2025). Global gender gap report 2025. World Economic Forum.

[22] (2023). Women in work 2023: closing the gender pay gap for good: a focus on the motherhood penalty. PricewaterhouseCoopers (PwC).

[23] Borges do Nascimento IJ, Abdulazeem HM, Weerasekara I, Marquez J, Vasanthan LT, Deeken G, Morgan R, Tan H, Yordi Aguirre I, Østeengaard L, Kularathne I, Azzopardi-Muscat N, van Kessel R, Martinez EZ, Permanand G, Novillo-Ortiz D (2025). Transforming women's health, empowerment, and gender equality with digital health: evidence-based policy and practice. Lancet Digit Health.

[24] (2025). Progress on the sustainable development goals: the gender snapshot 2025. United Nations Women, United Nations Department of Economic and Social Affairs.

[25] Figueroa CA, Luo T, Aguilera A, Lyles CR (2021). The need for feminist intersectionality in digital health. Lancet Digit Health.

[26] Lupton D, Maslen S (2019). How women use digital technologies for health: qualitative interview and focus group study. J Med Internet Res.

[27] Joshi A (2024). Big data and AI for gender equality in health: bias is a big challenge. Front Big Data.

[28] (2025). Informal carers. Australian Institute of Health and Welfare.

[29] (2024). Health workforce. Australian Institute of Health and Welfare.

[30] Lupton D (2019). The Australian Women and Digital Health Project: comprehensive report of findings. News and Media Research Centre.

[31] Organisation for Economic Co-operation and Development (2021). Development Co-operation Report 2021: Shaping a Just Digital Transformation.

[32] Yao R, Zhang W, Evans R, Cao G, Rui T, Shen L (2022). Inequities in health care services caused by the adoption of digital health technologies: scoping review. J Med Internet Res.

[33] Michie S, van Stralen MM, West R (2011). The behaviour change wheel: a new method for characterising and designing behaviour change interventions. Implement Sci.

[34] Davis FD (1989). Perceived usefulness, perceived ease of use, and user acceptance of information technology. MIS Q.

[35] Venkatesh V, Morris MG, Davis GB, Davis FD (2003). User acceptance of information technology: toward a unified view. MIS Q.

[36] (2022). Australian digital capability framework: version 1.0. Department of Employment and Workplace Relations, Australian Government.

[37] (2025). The complete statistical software for data science. StataCorp LLC.

[38] James C, Zevgitis L, Arabi S (2024). Gender diversity in Australia’s digital health workforce: special report 2024, 2nd ed.

[39] Thomas J, McCosker A, Parkinson S, Hegarty K, Featherstone D, Kennedy J, Holcombe-James I, Ormond-Parker L, Ganley L (2023). Measuring Australia’s Digital Divide: Australian Digital Inclusion Index 2023.

[40] Lin S, Duan L, Xu X, Cao H, Lu X, Wen X, Wei S (2025). Analyzing online search trends for kidney, prostate, and bladder cancers in China: infodemiology study using Baidu search data (2011-2023). JMIR Cancer.

[41] Wei S, Ma M, Wu C, Yu B, Jiang L, Wen X, Fu F, Shi M (2021). Using search trends to analyze web-based interest in lower urinary tract symptoms-related inquiries, diagnoses, and treatments in mainland China: infodemiology study of Baidu index data. J Med Internet Res.

[42] Wu PF, Summers C, Panesar A, Kaura A, Zhang L (2024). AI hesitancy and acceptability—perceptions of AI chatbots for chronic health management and long COVID support: survey study. JMIR Hum Factors.

[43] Nadarzynski T, Miles O, Cowie A, Ridge D (2019). Acceptability of artificial intelligence (AI)-led chatbot services in healthcare: a mixed-methods study. Digit Health.

[44] Mertz M, Toskovich K, Shields G, Attema G, Dumond J, Cameron E (2025). Exploring trust factors in AI-healthcare integration: a rapid review. Front Artif Intell.

[45] Limbu YB, Huhmann BA (2024). What influences consumers' online medication purchase intentions and behavior? A scoping review. Front Pharmacol.

[46] Ersöz S, Nissen A, Schütte R (2023). Risk, trust, and emotion in online pharmacy medication purchases: multimethod approach incorporating customer self-reports, facial expressions, and neural activation. JMIR Form Res.

[47] Michie S, Atkins L, West R (2014). The Behaviour Change Wheel: A Guide to Designing Interventions.

[48] Whitelaw S, Mamas MA, Topol E, Van Spall HGC (2020). Applications of digital technology in COVID-19 pandemic planning and response. Lancet Digit Health.

[49] Fujioka JK, Bickford J, Gritke J, Stamenova V, Jamieson T, Bhatia RS, Desveaux L (2021). Implementation strategies to improve engagement with a multi-institutional patient portal: multimethod study. J Med Internet Res.

[50] Fraccaro P, Vigo M, Balatsoukas P, Buchan I, Peek N, van der Veer SN (2017). Patient portal adoption rates: a systematic literature review and meta-analysis.

[51] (2019). I’d blush if I could: closing gender divides in digital skills through education. UNESCO & EQUALS.

