# Supplementary material for: Exploratory Analysis of the Digital Health Gender Paradox in Consumer-Facing Digital Health Technology Adoption: Cross-Sectional Survey Study
Source: JMIR Hum Factors. 2026 Sep 29;13:e101963. doi: 10.2196/101963 (PMC13662452; doi:10.2196/101963)
Supplement: Supplementary file 1 [file humanfactors_v13i1e101963_app1.pdf]

Australian consumers' behavioural study to understand the use of digital health in 2023

Thank you!

Let's start the survey

Consent Form - Declaration by Participant

I (the participant) have read and understood the Participant Information Statement. Any questions I had have been answered to my satisfaction. I agree that information provided by me or with my permission during the project may be included in a thesis, presentation and published in journals, and reports and articles published by Medibank on the condition that I cannot be identified.
I would like my information collected for this research study to be:

Would you like to be included in the $50 gift voucher draw?

◯ Yes
◯ No

Would like to be sent a copy of the outcomes of this research?

◯ Yes
◯ No

If your answer is "yes" to either of the above, please fill in the following

---

First Name

____

Last Name

____

Email Address

____

---

01 Age Category

◯ 18-24
◯ 25-29
◯ 30-34
◯ 35-39
◯ 40-44
◯ 45-49
◯ 50-54
◯ 55-59
◯ 60-64
◯ 65 and over

---

02 Gender

◯ Male
◯ Female
◯ Other

Please Specify

____

---

03 Select your state/ territory

◯ New South Wales
◯ Victoria
◯ Queensland
◯ Western Australia
◯ South Australia
◯ Tasmania
◯ Northern Territory
◯ Australian Capital Territory

04	Select your zone	◯ Metropolitan<br>◯ Rural<br>◯ Remote
05	Select your occupation	◯ Managers<br>◯ Professionals<br>◯ Technicians & Trades Workers<br>◯ Community & Personal Service Workers<br>◯ Clerical & Administrative Workers, Sales Workers, Labourers<br>◯ Machinery Operators & Drivers<br>◯ Other
	Please specify	__________________________________
06	Select your highest educational qualification	◯ Secondary Education<br>◯ Certificate Level<br>◯ Advanced Diploma & Diploma<br>◯ Bachelor Degree/Honours<br>◯ Graduate Certificate & Diploma<br>◯ Postgraduate Degree<br>◯ Other
	Please specify	__________________________________

7.1. Select your digital skills: Information and data literacy

		Agree	Neutral	Disagree	Do not know
1	I can browse and search information and digital content	◯	◯	◯	◯
2	I can filter data that I am searching for	◯	◯	◯	◯
3	I can collect data, information and digital content from trustworthy and reliable sources	◯	◯	◯	◯
4	I can manage my data, information and digital content	◯	◯	◯	◯

7.2 Rate your digital skills: Communication and collaboration

		Agree	Neutral	Disagree	Do not know
1	I can interact through digital technologies	○	○	○	○
2	I can share information through digital technologies	○	○	○	○
3	I can engage in citizenship through digital technologies	○	○	○	○
4	I can collaborate through digital technologies	○	○	○	○
5	I know the correct or acceptable way of using the internet (Netiquette)	○	○	○	○
6	I can manage my digital identity	○	○	○	○

7.3 Rate your digital skills: Digital content creation

		Agree	Neutral	Disagree	Do not know
1	I can develop digital content	◯	◯	◯	◯
2	I can integrate and re-elaborate digital content	◯	◯	◯	◯
3	I know about copyright and licenses matters	◯	◯	◯	◯
4	I know programming	◯	◯	◯	◯

7.4 Rate your digital skills: Safety

		Agree	Neutral	Disagree	Do not know
1	I can protect my devices	◯	◯	◯	◯
2	I can protect my personal data and privacy	◯	◯	◯	◯
3	I can protect health and well-being	◯	◯	◯	◯
4	I know how to protect my environment	◯	◯	◯	◯

7.5 Rate your digital skills: Problem Solving

		Agree	Neutral	Disagree	Do not know
1	I can solve technical problems in my digital devices	◯	◯	◯	◯
2	I can identify needs and technological responses	◯	◯	◯	◯
3	I can creatively use my digital technologies	◯	◯	◯	◯
4	I can identify my digital competence gaps	◯	◯	◯	◯

Research Aim 01:
Identifying the most preferred types of digital health technologies used by consumers.
What have been your preferred types of digital health technology in the last 12 months?

8.1 Have you had any "Telehealth" consultations?
- ◯ Yes
- ◯ No

If "yes", select the occasion you used it.
- ☐ Consultation with GP
- ☐ Hospital
- ☐ Psychology Consultation

How often you used it?
- ◯ Daily
- ◯ Weekly
- ◯ Monthly
- ◯ Less than 6 times per year
- ◯ Only once

If "No": please tell us why

____

8.2 Have you used any mobile health apps ?
- ◯ Yes
- ◯ No

If "yes"; Names of the mobile apps you used:

____

Indicate the service/s you gained
- ☐ To track activities/ exercise
- ☐ As a food & nutrition diary
- ☐ Make doctor appointments
- ☐ Online payments
- ☐ Store health records
- ☐ Track disease symptoms
- ☐ Disease diagnosis
- ☐ Health risk assessments
- ☐ Other

Please specify

____

How often you used these apps generally?
- ◯ Daily
- ◯ Weekly
- ◯ Monthly
- ◯ Less than 6 times per year
- ◯ Only once

If "No": Please tell us why?

____

8.3 Have you used any smart watches to mark vital signs such as heart rate & oxygen saturation etc or any other health wearble to get/track any health observations?
- ◯ Yes
- ◯ No

	If "yes"; name the wearble/s you have used.	________________________________
	If "yes" how often you used these wearables generally?	☐ Daily<br>☐ Weekly<br>☐ Monthly<br>☐ Less than 6 times per year<br>☐ Only Once
	Please tell us why.	________________________________
8.4	Have you used any chatbots/ virtual assistants ?	◯ Yes<br>◯ No
	If "yes", please indicate what services you use.	☐ Access health information<br>☐ Medical advices<br>☐ Make online appointments<br>☐ Make payments<br>☐ Symptom checking<br>☐ Disease diagnosis<br>☐ Access treatment plans<br>☐ Other
	Please specify	____
	If "Yes", How often did you use it?	◯ Daily<br>◯ Weekly<br>◯ Monthly<br>◯ Less than 6 times per year<br>◯ Only once
	Please tell us why?	____
8.5	Have you used any services like electronic prescriptions or online medicine ordering /delivering?	◯ Yes<br>◯ No
	Indicate the service	☐ Electronic prescriptions<br>☐ Online medicine ordering/ delivering
	If "yes", how often did you use it?	◯ Daily<br>◯ Weekly<br>◯ Monthly<br>◯ Less than 6 times per year<br>◯ Only once
	Please tell us why?	____

8.6 Do you currently use, know about or intend to use in the future, the following technologies?

		Current user	I'm aware of but do not use	Interested in using in future	Don't know
1	Smart glasses and any smart medical devices	○	○	○	○
2	Medical drones	○	○	○	○
3	Robot companions to assist my health	○	○	○	○
4	Health related games/ Patient engagement games	○	○	○	○
5	Virtual Reality/ Augmented Reality	○	○	○	○

8.6 Would you like to share your experience in using any of the above?

____

Research Aim: 02 The next set of questions will help us to understand the enablers, barriers, interests, choice influencers, and what starts and stops the usage of digital health technologies.

9.1 Capabilities

		Agree	Neutral	Disagree	Do not know
1	I am capable of using laptops or desktops	○	○	○	○
2	I am capable of using smart phones or tablets	○	○	○	○
3	I am capable of using smart watches	○	○	○	○
4	I am capable of using smart TVs	○	○	○	○
5	I can follow online instructions when using digital health tools.	○	○	○	○
6	I have physical disabilities like tremors when using smart phones/laptops/desktops/tablets/ smart watches	○	○	○	○
7	I prefer traditional health services (going to see doctor) rather than using digital health technology.	○	○	○	○
8	I have sensory disabilities like hearing /sight limitations when using smart phones/laptops/desktops/tablets/ smart watches.	○	○	○	○
9	I use regulatory approved (FDA, TGA) digital health devices only.	○	○	○	○
10	I am aware of some digital tools that can be used for my healthcare	○	○	○	○
11	I believe that smartphones, smart TVs, Tablets and Desktops are for communication and the general tasks only/ not for health service delivery.	○	○	○	○
12	I am familiar with existing regulations related to data privacy and sharing personal information when using digital tools	○	○	○	○

13

	I think there are benefits of using digital health tools in day-to-day life.	○	○	○	○
14	I know how to use digital tools to manage my health.	○	○	○	○
15	I can remember passwords and log in details easily when using digital health tools.	○	○	○	○

9.2 Opportunities

		Agree	Neutral	Disagree	Do not know
1	I have sufficient internet coverage in my area to access digital health tools.	○	○	○	○
2	There are digital health tools which are economically affordable for me.	○	○	○	○
3	I am supported/ convinced by healthcare professionals to use digital health tools.	○	○	○	○
4	I have many colleagues and family members who use digital health tools.	○	○	○	○
5	I have necessary devices that I need to manage my health using digital technologies	○	○	○	○

9.3 Motivations

		Agree	Neutral	Disagree	Do not know
1	My healthcare providers (GPs, Nurses & pharmacists) recommend me to use digital health tools.	○	○	○	○
2	I feel that my health data is secure while providing sensitive information.	○	○	○	○
3	I feel that my privacy is preserved while providing personal information.	○	○	○	○
4	I am interested in saving time by using telehealth and other remote patient monitoring systems.	○	○	○	○
5	I prefer e-prescription methods and online medicine purchasing and delivering facilities rather than going to the GPs..	○	○	○	○
6	I am interested in attending training sessions to develop my digital health skills.	○	○	○	○
7	I recommend digital health tools to my colleagues, friends and family.	○	○	○	○
8	I prefer to have online/computer - based copies of my pathology reports, prescriptions and other health records rather than paper records.	○	○	○	○
9	I want to have the ability to access all my health records anywhere any time through a smart device.	○	○	○	○
10	I am confident enough to use digital health technologies.	○	○	○	○
11	I become stressed and anxious when I experience technical issues while using digital tools.	○	○	○	○
12	I want to monitor my health daily using digital health tools.	○	○	○	○
13	I want to have digital health tools with language interpreter services.	○	○	○	○
