# Supplementary material for: Exploratory Analysis of the Digital Health Gender Paradox in Consumer-Facing Digital Health Technology Adoption: Cross-Sectional Survey Study
Source: JMIR Hum Factors. 2026 Sep 29;13:e101963. doi: 10.2196/101963 (PMC13662452; doi:10.2196/101963)
Supplement: Supplementary file 2 [file humanfactors_v13i1e101963_app2.docx]

**Table S1.** Exploratory gender interaction and stratified associations between COM-B domains and digital health technology use.

| Outcome & Predictor | Female × Predictor Interaction *aOR* (95% CI), *p*  *N*=416 | Women *aOR* (95% CI), *p*  *N*= 162 | Men *aOR* (95% CI), *p*  *N*=254 |
| --- | --- | --- | --- |
| Telehealth | | | |
| Digital literacy (z) | 1.19 (0.47–3.04), 0.712 | 0.51 (0.21–1.24), 0.136 | 0.56 (0.15–2.18), 0.407 |
| Capability (z) | 1.37 (0.54–3.45), 0.509 | 1.22 (0.55–2.68), 0.622 | 0.56 (0.14–2.29), 0.416 |
| Opportunity (z) | 0.91 (0.38–2.17), 0.830 | **1.55 (0.98–2.44), 0.060†** | 1.37 (0.34–5.46), 0.657 |
| Motivation (z) | 0.64 (0.27–1.55), 0.324 | 1.29 (0.67–2.47), 0.447 | **2.74 (0.89–8.48), 0.080†** |
| Mobile-health app | | | |
| Digital literacy (z) | 0.74 (0.41–1.34), 0.327 | 0.78 (0.41–1.50), 0.463 | 0.67 (0.27–1.66), 0.384 |
| Capability (z) | 0.60 (0.31–1.15), 0.126 | 0.92 (0.39–2.18), 0.848 | 1.59 (0.65–3.86), 0.306 |
| Opportunity (z) | 0.70 (0.37–1.30), 0.255 | **3.01 (1.66–5.48), < 0.001** | **3.76 (1.94–7.31), <0.001** |
| Motivation (z) | 1.29 (0.76–2.18), 0.341 | 1.14 (0.78–1.64), 0.502 | 0.75 (0.45–1.25), 0.266 |
| Wearable device | | | |
| Digital literacy (z) | 1.19 (0.64–2.20), 0.588 | 0.68 (0.32–1.44), 0.316 | 1.24 (0.51–3.03), 0.634 |
| Capability (z) | 1.63 (0.89–2.99), 0.116^†^ | 1.41 (0.71–2.78), 0.321 | **0.23 (0.09–0.59), 0.002** |
| Opportunity (z) | 0.97 (0.54–1.74), 0.915 | **1.52 (1.01–2.31), 0.046** | **2.89 (1.17–7.17), 0.022** |
| Motivation (z) | 1.06 (0.55–2.07), 0.855 | 1.00 (0.65–1.53), 0.986 | 1.26 (0.60–2.64), 0.538 |
| Chatbot / virtual assistant | | | |
| Digital literacy (z) | **2.10 (1.12–3.96), 0.021** | 1.35 (0.74–2.46), 0.331 | 0.75 (0.25–2.21), 0.600 |
| Capability (z) | **1.83 (1.02–3.29), 0.044** | 0.75 (0.40–1.39), 0.354 | **0.34 (0.13–0.88), 0.026** |
| Opportunity (z) | 1.34 (0.73–2.45), 0.350 | 0.96 (0.63–1.48), 0.866 | 1.33 (0.61–2.94), 0.474 |
| Motivation (z) | 1.42 (0.77–2.63), 0.262 | 1.26 (0.86–1.84), 0.238 | 1.39 (0.68–2.83), 0.363 |
| e-Pharmacy | | | |
| Digital literacy (z) | 1.55 (0.68–3.53), 0.295 | 0.99 (0.56–1.78), 0.986 | 1.06 (0.29–3.96), 0.928 |
| Capability (z) | 1.65 (0.79–3.46), 0.184 | 0.96 (0.52–1.78), 0.897 | 0.36 (0.11–1.24), 0.105^†^ |
| Opportunity (z) | 1.37 (0.70–2.70), 0.358 | 1.17 (0.79–1.74), 0.438 | 1.00 (0.41–2.43), 0.992 |
| Motivation (z) | 1.14 (0.51–2.53), 0.748 | 1.37 (0.93–2.02), 0.117 | 2.20 (0.81–5.97), 0.121 |

Note. Each model adjusts for age, education, occupation, region, and rural/remote residence. All predictors were standardised (*z*-scores), so each odds ratio (*OR*) represents the change in odds for a one-standard-deviation increase in the predictor. † *p* = 0.05–0.10 (trend). Bold indicates statistically significant results at *p* < 0.05.

**Table S2.** Item-level mapping, scoring, and missingness of the theory-informed COM-B composite indices.

| **Composite** | **Proposed COM-B subdomain** | **Survey item** | **Reverse-coded** | **Do not know, n (%)** | **Missing, n (%)** | **Conceptual note** |
| --- | --- | --- | --- | --- | --- | --- |
| Capability | Psychological capability | I am capable of using laptops or desktops | No | 0 (0.00) | 0 (0.00) | Digital skill |
| Capability | Psychological capability | I am capable of using smartphones or tablets | No | 0 (0.00) | 0 (0.00) | Digital skill |
| Capability | Psychological capability | I am capable of using smartwatches | No | 9 (2.16) | 0 (0.00) | Digital skill |
| Capability | Psychological capability | I am capable of using smart TVs | No | 3 (0.72) | 0 (0.00) | Digital skill |
| Capability | Psychological capability | I can follow online instructions when using digital health tools | No | 2 (0.48) | 0 (0.00) | Digital skill/knowledge |
| Capability | Physical capability | I have physical disabilities such as tremors when using digital devices | Yes | 6 (1.44) | 0 (0.00) | Physical limitation |
| Capability | Psychological capability† | I prefer traditional health services rather than using digital health technology | Yes | 10 (2.40) | 0 (0.00) | Also conceptually related to reflective motivation |
| Capability | Physical capability | I have sensory disabilities such as hearing/sight limitations when using digital devices | Yes | 14 (3.37) | 0 (0.00) | Physical/sensory limitation |
| Capability | Psychological capability† | I use regulatory-approved (FDA/TGA) digital health devices only | No | 40 (9.62) | 0 (0.00) | Knowledge/choice; possible reflective-motivation overlap |
| Capability | Psychological capability | I am aware of digital tools that can be used for my healthcare | No | 12 (2.88) | 0 (0.00) | Awareness/knowledge |
| Capability | Psychological capability† | I believe smartphones, smart TVs, tablets and desktops are for communication/general tasks only, not health-service delivery | Yes | 4 (0.96) | 0 (0.00) | Knowledge/belief; possible reflective-motivation overlap |
| Capability | Psychological capability | I am familiar with regulations related to data privacy and sharing personal information when using digital tools | No | 8 (1.92) | 0 (0.00) | Knowledge |
| Capability | Psychological capability† | I think there are benefits of using digital health tools in day-to-day life | No | 7 (1.68) | 0 (0.00) | Perceived benefit; also related to reflective motivation |
| Capability | Psychological capability | I know how to use digital tools to manage my health | No | 12 (2.88) | 0 (0.00) | Knowledge/skill |
| Capability | Psychological capability | I can remember passwords and login details easily when using digital health tools | No | 4 (0.96) | 0 (0.00) | Cognitive skill |
| Opportunity | Physical opportunity | I have sufficient internet coverage in my area to access digital health tools | No | 5 (1.20) | 0 (0.00) | Infrastructure/access |
| Opportunity | Physical opportunity | There are digital health tools which are economically affordable for me | No | 31 (7.45) | 0 (0.00) | Financial/material access |
| Opportunity | Social opportunity | I am supported/convinced by healthcare professionals to use digital health tools | No | 11 (2.64) | 0 (0.00) | Professional/social support |
| Opportunity | Social opportunity | I have many colleagues and family members who use digital health tools | No | 19 (4.57) | 0 (0.00) | Social environment |
| Opportunity | Physical opportunity | I have necessary devices that I need to manage my health using digital technologies | No | 16 (3.85) | 0 (0.00) | Material/device access |
| Motivation | Reflective motivation† | My healthcare providers recommend me to use digital health tools | No | 4 (0.96) | 0 (0.00) | Professional influence; also related to social opportunity |
| Motivation | Reflective motivation | I feel that my health data is secure while providing sensitive information | No | 17 (4.09) | 0 (0.00) | Trust/security belief |
| Motivation | Reflective motivation | I feel that my privacy is preserved while providing personal information | No | 17 (4.09) | 0 (0.00) | Privacy/trust belief |
| Motivation | Reflective motivation | I am interested in saving time by using telehealth and other remote patient monitoring systems | No | 3 (0.72) | 0 (0.00) | Expected benefit/interest |
| Motivation | Reflective motivation | I prefer e-prescription methods and online medicine purchasing/delivery rather than going to GPs | No | 4 (0.96) | 0 (0.00) | Preference |
| Motivation | Reflective motivation | I am interested in attending training sessions to develop my digital health skills | No | 3 (0.72) | 0 (0.00) | Intention/interest |
| Motivation | Reflective motivation | I recommend digital health tools to my colleagues, friends and family | No | 10 (2.40) | 0 (0.00) | Positive evaluation/intention |
| Motivation | Reflective motivation | I prefer online/computer-based copies of pathology reports, prescriptions and other health records rather than paper records | No | 3 (0.72) | 0 (0.00) | Preference |
| Motivation | Reflective motivation | I want the ability to access all my health records anywhere and anytime through a smart device | No | 5 (1.20) | 0 (0.00) | Intention/preference |
| Motivation | Reflective motivation† | I am confident enough to use digital health technologies | No | 6 (1.44) | 0 (0.00) | Self-efficacy; also related to psychological capability |
| Motivation | Automatic motivation | I become stressed and anxious when I experience technical issues while using digital tools | Yes | 6 (1.44) | 0 (0.00) | Emotional response |
| Motivation | Reflective motivation | I want to monitor my health daily using digital health tools | No | 8 (1.92) | 0 (0.00) | Intention |
| Motivation | Reflective motivation† | I want digital health tools with language interpreter services | No | 11 (2.64) | 0 (0.00) | Preference reflecting an opportunity/access need |

Note: COM-B = Capability, Opportunity, Motivation–Behaviour. Items are presented according to their original survey grouping. Proposed COM-B subdomain mappings were applied to improve conceptual transparency and should not be interpreted as independently validated subscales. Items marked † have plausible conceptual relevance to more than one COM-B domain, as indicated in the conceptual-note column. Original response categories were Do not know, Disagree, Neutral, and Agree, coded 1–4 in the primary analysis. Negatively worded items were reverse-coded so that higher composite scores represented greater Capability, Opportunity, or Motivation. Composite scores were calculated as the mean of their constituent items. There was no true item-level missingness across the 33 COM-B items; “Do not know” responses are therefore reported separately from missing data.

**Table S3.** Exploratory measurement properties of the COM-B composites.

| **Composite** | **Items** | **Cronbach α** | **KMO** | **Factors with eigenvalue >1** | **Cumulative variance (%)** |
| --- | --- | --- | --- | --- | --- |
| Capability | 15 | .83 | .89 | 3 | 61.4 |
| Opportunity | 5 | .76 | .79 | 1 | 51.3 |
| Motivation | 13 | .61 | .61 | 5 | 57.1 |

Note. KMO = Kaiser–Meyer–Olkin measure of sampling adequacy. Exploratory factor analyses were undertaken to examine the dimensionality of the investigator-constructed, theory-informed composites rather than to validate a three-factor COM-B measurement model. Factors with eigenvalues >1 are reported descriptively and should not be interpreted as validated subscales.

**Table S4.** Item-total statistics and internal consistency of the Motivation composite.

| Motivation item | Corrected item-rest correlation | Cronbach α if item deleted |
| --- | --- | --- |
| Healthcare-provider recommendation/support | .185 | .602 |
| Health-data security | .239 | .595 |
| Privacy protection | .352 | .569 |
| Time-saving | .261 | .590 |
| E-pharmacy preference | **.140** | **.610** |
| Training interest | .208 | .599 |
| Recommendation to friends/family | .300 | .581 |
| Electronic-record preference | .263 | .589 |
| Access to health data | .222 | .596 |
| Confidence using digital health technology | .378 | .568 |
| Stress/anxiety with digital technology† | .271 | .587 |
| Intention to monitor health | .293 | .583 |
| Language-support preference | .175 | .606 |

Note. Overall Cronbach α=.610. †Reverse-coded before construction of the Motivation composite. Corrected item-rest correlations represent correlations between each item and the composite formed from the remaining items.

**Table S5.** Sensitivity analyses of the association between Motivation and e-pharmacy use.

| **Motivation specification** | **No. of items** | **aOR (95% CI)** | ***p* value** | **Pseudo R²** |
| --- | --- | --- | --- | --- |
| Original Motivation composite | 13 | 1.50 (1.05-2.15) | .026 | .169 |
| Excluding e-pharmacy preference item | 12 | 1.51 (1.05-2.16) | .026 | .169 |
| Reduced Motivation composite† | 10 | 1.46 (0.99–2.15) | .058 | .165 |

Note. aOR = adjusted odds ratio; CI = confidence interval. All models used the same specification as the primary e-pharmacy model and adjusted for gender, digital literacy, Capability, Opportunity, age, education, occupation, rural/remote residence, and region. The original Motivation composite contained 13 items. The first sensitivity analysis excluded the e-pharmacy preference item to assess potential conceptual overlap between the predictor and outcome. †The reduced 10-item composite excluded the three items with the lowest corrected item-rest correlations: e-pharmacy preference, language-support preference, and healthcare-provider recommendation/support. All predictors were standardised (z-scores).

**Table S6.** COM-B-directed coding framework for open-text reasons for non-use of digital health technologies.

| **Technology** | **COM-B domain** | **Meaning** | **Example statements/triggers** |
| --- | --- | --- | --- |
| Telehealth | Capability | Lack of knowledge/awareness | “I don’t know about them”; “I don’t even know what this is about” |
|  | Opportunity | Not offered/access constraints | “Not offered”; “not provided with the option”; “They haven’t been offered” |
|  | Motivation | Preference/lack of perceived need | “Prefer face-to-face”; “I have not needed to”; “no need at this stage” |
| Mobile-health app | Capability | Lack of skill/understanding | “how to operate”; “not good at”; “too complicated” |
|  | Opportunity | Access/cost/not offered | “never been offered”; “cost”; “nobody tell me” |
|  | Motivation | Preference/interest/trust | “prefer human”; “not interested”; “don’t trust”; “not reliable” |
| Wearable device | Capability | Skill/usability limitations | “too complicated”; “too hard to do”; “don’t know [how]” |
|  | Opportunity | Cost/device ownership | “too expensive”; “can’t afford”; “I don’t own a smart watch” |
|  | Motivation | Preference/lack of perceived need | “no need”; “not interested”; “don’t want”; “don’t see the merit” |
| Chatbot/virtual assistant | Capability | Awareness/know-how gaps | “how to use”; “do not understand”; “not aware” |
|  | Opportunity | Not available/offered/encountered | “never been offered”; “no access”; “not available”; “haven’t come across any” |
|  | Motivation | Preference/trust/perceived utility | “prefer human”; “don’t trust”; “frustrating”; “useless”; “not needed” |
| e-Pharmacy | Capability | Knowledge/skill/confidence | “lack of information”; “not confident”; “don’t understand”; “don’t know how” |
|  | Opportunity | Lack of access/offer/opportunity | “haven’t had the opportunity”; “not offered”; “no access” |
|  | Motivation | Preference/trust/safety | “prefer in person”; “don’t trust”; “not safe”; “didn’t find [it] necessary” |
