# Supplementary material for: Exploratory Analysis of the Digital Health Gender Paradox in Consumer-Facing Digital Health Technology Adoption: Cross-Sectional Survey Study
Source: JMIR Hum Factors. 2026 Sep 29;13:e101963. doi: 10.2196/101963 (PMC13662452; doi:10.2196/101963)
Supplement: Supplementary file 3 [file humanfactors_v13i1e101963_app3.docx]

**Table S1.** Summary of self-reported barriers to digital health technology adoption by COM-B^a^ domain.

| Technology | Capability (skill/confidence) | Opportunity (access/offer/logistics) | Motivation (trust/need/preference) |
| --- | --- | --- | --- |
| Telehealth | Limited awareness or knowledge of how telehealth works. | Not offered the option; lack of time or suitable appointment pathways. | Preference for face-to-face care; low perceived need. |
| Mobile-health app | Limited technical skills; overwhelmed by number of apps. | Not offered or aware; cost and access barriers. | Preference for human interaction; lack of interest or trust in accuracy. |
| Wearable device | Difficulty operating devices; low digital confidence. | Cost, lack of ownership, or device unavailability. | No perceived need; privacy or identity concerns; prefer not to track. |
| Chatbot/virtual assistant | Lack of knowledge on use; uncertainty about function. | Not encountered in health settings; limited availability or affordability. | Prefer human contact; low trust; perceive chatbots as inaccurate or unhelpful. |
| E-pharmacy | Unfamiliarity with online ordering; low confidence. | Not on medication or not offered e-pharmacy options. | Prefer in-person purchasing; distrust of online safety or privacy. |

^a^COM-B: Capability, Opportunity, Motivation – Behaviour.
